# Trauma Quality Indicators’ usage limitations in severe trauma patients

**DOI:** 10.1590/0100-6991e-20202769

**Published:** 2021-02-18

**Authors:** PEDRO DE SOUZA LUCARELLI ANTUNES, PAULA RIBEIRO LIBÓRIO, GIOVANNA MENNITTI SHIMODA, LUCA GIOVANNI ANTONIO PIVETTA, JOSÉ GUSTAVO PARREIRA, JOSE CESAR ASSEF

**Affiliations:** 1 - Faculdade de Ciências Médicas da Santa Casa de São Paulo, Disciplina de Cirurgia - São Paulo - SP - Brasil; 2 - Irmandade da Santa Casa de Misericórdia de São Paulo, Serviço de Emergência - São Paulo - SP - Brasil

**Keywords:** Traumatology, Multiple Trauma, Trauma Severity Indices, Quality of Health Care, Traumatologia, Traumatismo Múltiplo, Índices de Gravidade do Trauma, Qualidade da Assistência à Saúde

## Abstract

**Purpose:**

to analyze the relation between Trauma Quality Indicators (QI) and death, as well as clinical adverse events in severe trauma patients.

**Methods:**

analysis of data collected in the Trauma Register between 2014-2015, including patients with Injury Severity Score (ISS) > 16, reviewing the QI: (F1) Acute subdural hematoma drainage > 4 hours with Glasgow Coma Scale (GCS) <9; (F2) emergency room transference without definitive airway and GCS <9; (F3) Re-intubation within 48 hours; (F4) Admission-laparotomy time greater than 60 min in hemodynamically instable patients with abdominal bleeding; (F5) Unprogrammed reoperation; (F6) Laparotomy after 4 hours; (F7) Unfixed femur diaphyseal fracture; (F8) Non-operative treatment for abdominal gunshot; (F9) Admission-tibial exposure fracture treatment time > 6 hours; (F10) Surgery > 24 hours. T the chi-squared and Fisher tests were used to calculate statistical relevance, considering p<0.05 as relevant.

**Results:**

127 patients were included, whose ISS ranged from 17 to 75 (28.8 + 11.5). There were adverse events in 80 cases (63%) and 29 died (22.8%). Twenty-six patients had some QI compromised (20.6%). From the 101 patients with no QI, 22% died, and 7 of 26 patients with compromised QI (26.9%) (p=0.595). From the patients with no compromised QI, 62% presented some adverse event. From the patients with any compromised QI, 18 (65.4%) had some adverse event on clinical evolution (p=0.751).

**Conclusion:**

the QI should not be used as death or adverse events predictors in severe trauma patients.

## RESUMO

**Objetivo:**

analisar relação entre comprometimento de Filtros de Qualidade (FQ) com complicações e mortalidade entre vítimas de trauma grave.

**Métodos:**

análise dos dados coletados para o Registro de Trauma entre 2014 e 2015, sendo incluídos os traumatizados com Injury Severity Score (ISS) > 16 e analisados os FQ: (F1) drenagem de Hematoma Subdural Agudo (HSA) > 4 horas com Escala de Coma de Glasgow (ECG) <9, (F2) transferência da sala de emergência sem via aérea definitiva e com ECG<9, (F3) reintubação traqueal em até 48 horas, (F4) tempo entre admissão e laparotomia exploradora maior que 60 minutos em pacientes instáveis com foco abdominal, (F5) reoperação não programada, (F6) laparotomia > 4 horas, (F7) fratura de diáfise de fêmur não fixada, (F8) tratamento não operatório em Ferimento por Arma de Fogo (FAF) abdominal, (F9) tempo entre admissão e tratamento de fraturas expostas de tíbia > 6 horas, (F10) operação > 24 horas. Testes de Chi quadrado e Fisher para a análise estatística, considerando significativo p<0,05, foram usados.

**Resultado:**

foram incluídos 127 pacientes com ISS entre 17 a 75 (28,8 + 11,5). As complicações ocorreram em 80 casos (63%) e 29 morreram (22,8%). Vinte e seis pacientes apresentaram algum FQ comprometido (20,6%). Dos 101 doentes sem FQ comprometido, 22% faleceram, o que ocorreu em 7 dos 26 doentes com comprometimento dos FQ (26,9%) (p=0,595). Dos doentes sem FQ comprometido, 62% tiveram alguma complicação. Entre os pacientes com FQ comprometido, 18 (65,4%) tiveram complicações (p=0,751).

**Conclusão:**

os FQs não devem ser utilizados como preditor de mortes ou complicações evitáveis nas vítimas de traumas graves.

## INTRODUCTION

Trauma is a serious public health issue, especially in large urban centers, being the third leading cause of death in the world^1^. It is the pathological process resulting from sudden energy exchanges in different body segments, caused by agents of varying etiology, nature, and extent^2^.

From a broader perspective on all the phases that compose this disease, it is necessary to consider, in addition to pre- and intra-hospital care, predisposing factors of socioeconomic and cultural nature, as well as events that can be avoided through prevention^3^. Attention should also be directed to the consequences, regarding temporary and permanent sequelae, which are related to the quality of care for traumatized patients.

In this context, with the objective of improving patients’ prognosis, it is possible to develop quality programs that continuously monitor the elements of diagnosis, treatment, and evolution of victims^4^. Several models of quality programs to trauma care have been proposed, such as morbidity and mortality meetings, study of avoidable deaths, auditing monitoring of indicators, establishment of morbidity and mortality review committees, cycle closing with the team, and especially trauma records. The use of trauma indices, such as the Glasgow Coma Scale (GCS)^5^, the Abbreviated Injury Scale (AIS)^6^ and the Injury Severity Score (ISS)^7^ to stratify the severity of the victims, associated with the monitoring of quality/auditing indicators (QIs), proved to be effective in identifying potentially preventable deaths^8^, allowing detailing of improvement points in care^9^.

In this scenario, the victims of high-energy trauma, considered severe by trauma indices, are those with the greatest immediate and late impact, represented by death, as well as in-hospital complications, and permanent sequelae. Thus, the study of severe patients becomes essential to aim for points of improvement in outcomes.

The aim of this study is to analyze the usefulness of different Quality Indicators, based on those proposed by the American College of Surgeons^10^, as parameters of treatment effectiveness and improvement of prognosis in the care of severely injured victims (ISS> 16) in a Service Specialized in Trauma Care.

## METHODS

This study was submitted to the institution’s Ethics in Research Committee and approved under protocol number CAAE 30831214.4.0000.5479. We conducted a retrospective analysis of the Trauma Registry data, which included trauma patients considered severe, with Injury Severity Score (ISS) > 16, admitted between 2014 and 2015 (12 months) in the Emergency Room of the Irmandade da Santa Casa de Misericordia de São Paulo.

### Database

The recording of information on traumatized patients is part of the Trauma Registry of the Emergency Room of Irmandade da Santa Casa de Misericordia de São Paulo^11^, pertaining to the Traumatized Care Quality Program. Data were collected in the Emergency Room and stored in a software specifically developed for this purpose (iTreg - ECOssistemas) during hospitalization under the care of the Surgery Department.

### Data analysis

We reviewed data on trauma victims whose information was in the database. For the purposes of statistical analysis, we stratified injuries’ severity with the AIS. The inclusion criteria for the research was an ISS > 16. We collected data on demographics, identified injuries, treatment, complications, and deaths.

Based on the QIs idealized by the American College of Surgeons and the Brazilian Society for Integral Assistance to Traumatized Patients (SBAIT), we proposed the analysis of the occurrence (positivity meaning compromise) of the following indicators:

(QI1) Time between admission and drainage of acute subdural hematoma (ASH) greater than 4 hours in patients with GCS < 9;
(QI2) Transfer from the emergency room without definitive airway and GCS < 9;
(QI3) Reintubation within 48 hours of extubation;
(QI4) Time between admission and exploratory laparotomy greater than 60 minutes in unstable patients with abdominal focus;
(QI5) Unscheduled reoperation;
(QI6) Time between admission and laparotomy greater than 4 hours;
(QI7) Non-fixed femoral diaphysis fracture;
(QI8) Nonoperative treatment of abdominal gunshot wound (GSW);
(QI9) Time between admission and treatment of exposed tibial fractures greater than 6 hours; and
(QI10) Time between admission and surgery greater than 24 hours.

We analyzed the relationship between the occurrence of the indicators and demographic data, identified injuries, treatments, complications, and deaths. We performed statistical analysis using the Chi-square and the Fisher tests, given the qualitative nature of the variables evaluated, with p<0.05 considered significant.

## RESULTS

We analyzed 127 patients, aged between 14 and 92 years (40.5 ± 18.6 years). Among them, 77.9% sustained blunt trauma, the others being victims of stabbing wounds. Table 1 describes the patients’ demographics in detail. Complications occurred in 80 cases (63%), respiratory infection (33.9%) and sepsis (41.7%) being the most frequent. Twenty-nine patients died (22.8%), the most common cause being Traumatic Brain Injury (TBI), in 18 patients (62.1%). Secondary infections (13) and hemorrhage (2) also contributed as the cause of death for patients, with four patients presenting infection secondary to TBI.

**Table 1:** Detailed patients’ demographic data.

Blunt Trauma mechanism	Occurrence (patients)				
Four-wheel vehicle crash	10	With seat belt	5	Trapped in wreckage	3
		Without seat belt	5	Not trapped in wreckage	7
Motorcycle crash	19	With helmet	15		
		Without helmet	4		
Trampling	31				
Bicycle crash	2				
Fall	22	Higher than 1.5m	17		
		Lower than 1.5m	5		
Fall from standing height	4				
Assault	6				
Other	5				
Associated conditions					
Drugs intoxication	16				
Alcohol intoxication	18				
Arterial Hypertension	11				
Smoking	5				
Diabetes Mellitus	3				
Solid Neoplasm	1				
HIV infection	2				
Pre-hospital Care					
Directly from scene	113				
Other	14	Emergency referral	5		
		Other	9		
Pre-hospital support	Present	40	Sedation + Tracheal intubation	15	
	Absent	87			
ER care					
Tracheal Intubation	10				
Thorax X-Ray	96	normal	55		
		abnormal	41		
Pelvis X-Ray	69	normal	60		
		abnormal	9		
FAST	64	normal	47		
		abnormal	17		
Image					
Cranial CT	94	normal	41		
		abnormal	53		
Cervical CT	68	normal	64		
		abnormal	4		
Face CT	28	normal	16		
		abnormal	12		
Thorax CT	74	normal	33		
		abnormal	41		
Abdominal CT	75	normal	35		
		abnormal	40		
Arteriography	15	normal	8		
		embolization	7		
Injuries					
Head					
	Epidural hematoma	5			
	Subdural hematoma	5			
	Cerebral contusion	27			
	Subarachnoid hemorrhage	14			
	Diffuse Axonal Injury	11			
	Edema	8			
Cervical					
	Vertebral Fracture	1			
	Internal Jugular Vein injury	3			
	Laryngeal injury	1			
Face					
	Bone Fracture	11			
Thorax					
	Hemopneumothorax	26	Pneumothorax	4	
			Hemothorax	16	
			Both	6	
	Rib fracture	25	Stable	10	
			Flail-chest	15	
	Sternal fracture	3			
	Subclavian vascular injury	2			
	Heart injury	3			
	Diaphragmatic injury	10			
	Lung injury	32			
	Thoracic aorta injury	4			
					
Abdominal					
	Liver injury	21			
	Spleen injury	19			
	Kidney injury	14			
	Small bowel injury	6	Duodenal	2	
			Not duodenal	4	
	Colon injury	5			
	Stomach injury	3			
	Pancreatic injury	3			
	IVC injury	2			
	Bladder injury	1			
Extremity and Pelvis					
	Closed fractures	33	Upper limbs	18	
			Lower limbs	15	
	Open fractures	10	Upper limbs	6	
			Lower limbs	4	
	Pelvic fracture	11	Stable	7	
			Unstable	4	
	Spine fracture		Cervical	6	
			Thoracic	12	
			Lumbar	3	
Treatment					
Surgery	79	Craniotomy	11		
		Cervicotomy	2		
		Thoracotomy	10		
		Thoracoscopy	4		
		Laparotomy	31	Primary repair	27
				Damage control	4
		Laparoscopy	3		
		Limb surgical fixation	16		
Non-surgical treatment	48	Abdominal Non-Operative Treatment protocol	22		
		Other	26		
Complications					
Respiratory Insufficiency	20				
ARDS	2				
Non-planned re-operation	1				
Iatrogenical pneuothorax	3				
Iatrogenical Hemothorax	2				
Retained Hemothorax	6				
DVT	1				
AKI	13				
Digestive fistula	4				
Intrabdominal Hypertension	2				
Rabdomyolysis	11				
Pressure ulcers	6				
Infection	65	Respiratory	43		
		Urinary	5		
		Sepsis (blood)	5		
		Intra-abdominal	5		
		Subcutaneous	3		
		Pleural	3		
		Phlebitis	1		
Sepsis	53				
Death	29	TBI	18	Alone	14
				Associated with infection	4
		Hemorrhage	2		
		Infection	13		

FAST- Focused Assessment with Sonography for Trauma; CT- Computerized Tomography; IVC - Inferior Vena Cava; ARDS - Acute Respiratory Distress Syndrome; DVT - Deep Vein Thrombosis; AKI - Acute Kidney Injury; TBI - Traumatic Brain Injury.

**Graph 1:**
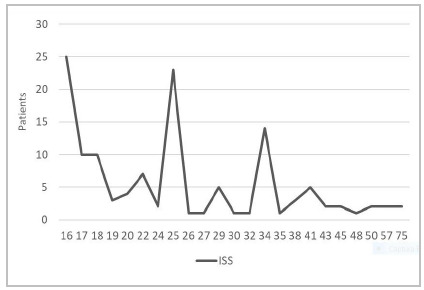
Patients’ severity bases on Injurity Severity Score (ISS).

**Graph 2:**
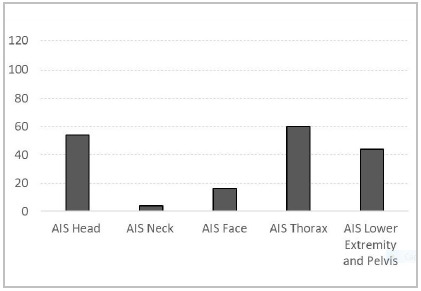
Pattern of body region’s injuries in the patients, based on AIS.

The ISS ranged from 17 to 75 (mean 26.3 ± 11.5) (Graph 1). As for segmental injuries, represented by AIS values > 0 in each traumatized body segment, 54 patients had injuries in the head segment, four in the neck, 16 in the face, 60 in the chest, and 44 in the limbs and pelvis (Graph 2).

**Graph 3:**
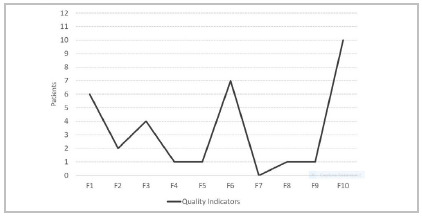
Trauma Quality Indicators commitment distribution, based on occurrence.

Twenty-six patients had some compromised quality indicator (20.5%). Ten patients (7.9%) had a compromised QI10 (surgery > 24h), this being the most prevalently affected QI (38.5% of the occurrences). Seven patients (5.5%) had a nonconformant QI6 (laparotomy > 4h - 26.9%), and 6 patients (4.7%) displayed a QI1 (ASH drainage > 4h + GCS > 9 - 23.1%). Only three patients underwent surgery between 4 and 24 hours, one neurosurgical approach and two laparotomies (Graph 3).

**Graph 4:**
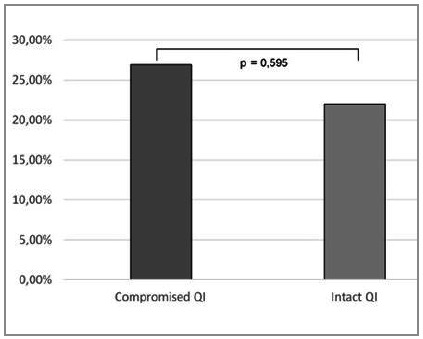
Comparative analysis of death among patients with compromised Trauma Quality Indicators and those with no commitment. The graph shows death in 22% of patients with no QI commitment, as well as in 26.9% of patients with compromised QI. This difference was not statistically significant (p=0.595).

**Graph 5:**
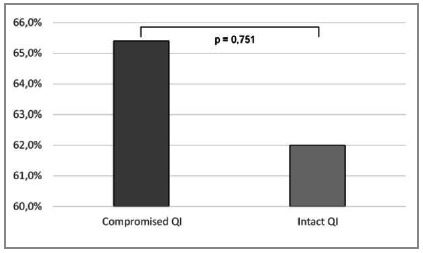
Assessment of complications and compromised of QIs.

Of the 101 patients who showed no compromised QIs, 22% died and 62% sustained some complication, which occurred respectively in 26.9% (7/26) and 65.4% (18/26) of patients with some compromised QI (Graph 4 and 5 - p=0.595 and p=0.751, respectively).

## DISCUSSION

According to the literature, greater severity of the identified injuries is frequent in blunt trauma^12^ ^,^ ^13^, a fact corroborated by the results of this study. As for complications, like other studies^14^, we noted the prevalence of respiratory tract infections and sepsis. However, the highest mortality was concentrated in patients who suffered traumatic brain injury, which is justified by the exchange of energy involved in the mechanism of this type of trauma, causing injuries in the short and long term, sometimes irreversible^15^.

Regarding the commitment of Quality Indicators, which occurred in about one of every five patients with ISS > 16, it was mostly related to the time between admission and surgery, especially laparotomy. However, despite the QIs nonconformity in patients with severe trauma, this situation has not been able to significantly impact prognosis and mortality. Specifically, the complications rate was 65.4% when QIs were compromised, as opposed to 62% in the absence of any compromise. Deaths occurred in 26.9% in the subgroup with QIs noncompliance and 22% when they were adhered to. From this data we can infer that, when it comes to patients with high ISS, ie victims of multiple injuries of greater severity, the use of Quality Indicators is not effective in identifying flaws in trauma care. This fact occurs mainly because, unlike other populations, the degree of organic impairment caused by injury is so high that even respecting care standards proposed by QIs cannot reverse the condition or significantly improve prognosis.

Our findings are different from those found in previous studies related to the use of QIs in the identification of preventable complications and deaths^8^ ^,^ ^9^, whose results demonstrated improvement in the quality of care and in the outcome when the pre-established indicators were complied with. This difference suggests that pre-hospital care and surgical conduct strategies are the main factors related to mortality of patients with severe injuries^16^ ^,^ ^17^. Moreover, since the QIs are idealized to identify preventable deaths and complications, they remain statistically unchanged.

Therefore, we can conclude that, in this study, the QIs were not good parameters to assess the quality of care for severe trauma, since most deaths in severe trauma patients were not preventable. This implies the development of specific approaches to assess the mortality of severe trauma patients, which should be focused on prevention strategies, pre-hospital care, and damage control^16^ ^,^ ^18^.

On the other hand, when conceiving Traumatized Care Quality Programs, those who use QIs to identify points of improvement have a bias factor in severe trauma patients. Such individuals must be analyzed more thoroughly, apart from other trauma victims. This would bring benefits both to the severely traumatized, whose selection should be more comprehensive, with extra attention, and to the mildly and moderately injured. In the latter, QIs can indicate those whose care process must be reviewed, without risk of selection bias. Such findings may improve the approach strategies to trauma patients of every severity level.
